# Evaluation of body mass index as a prognostic indicator from two rough‐toothed dolphin (*Steno bredanensis*) mass strandings in Florida

**DOI:** 10.1002/ece3.5574

**Published:** 2019-08-23

**Authors:** Bree L. Karns, Ruth Y. Ewing, Adam M. Schaefer

**Affiliations:** ^1^ University of Miami Rosenstiel School of Marine and Atmospheric Science Miami FL USA; ^2^ Marine Mammal Health and Stranding Response Program National Marine Fisheries Service National Oceanic and Atmospheric Administration Miami FL USA; ^3^ Harbor Branch Oceanographic Institute Florida Atlantic University Ft Pierce FL USA

**Keywords:** body mass index, marine mammal rehabilitation, mass strandings, rough‐toothed dolphins

## Abstract

Rough‐toothed dolphins (*Steno bredanensis*) are a common mass stranding species in Florida. These large stranding events typically include a small number of sick or injured individuals and a much larger number of healthy individuals, making rapid triage essential. Little data exist on rehabilitation outcomes, and historically, successful outcomes are limited. Furthermore, very little data exist on the feeding habits and dietary needs of this species. This study compared morphology and body mass index (BMI) in two rough‐toothed dolphin mass stranding events in Florida: August 2004 (*n* = 36) and March 2005 (*n* = 32). The two groups were significantly different in morphologic measurements, with age and gender‐adjusted intake BMI significantly (*p* < .01) different (2004 = 0.34 ± 0.02; 2005 = 0.41 ± 0.02) between groups. Ten animals from 2005 had weights tracked throughout the rehabilitation process and demonstrated an initial drop in BMI followed by an increase and a plateau prior to release. When comparing initial BMI by stranding outcome, individuals that were rehabilitated and released had a significantly (*p* = .03) higher BMI than individuals who were euthanized. However, there was no difference between dolphins that died of natural causes (*p* = .56) and animals successfully rehabilitated. Analysis of BMI can be a useful marker in triage during a stranding, when resources are limited to identify individuals most likely to survive, as well as in determining the appropriate body condition for release. The data reported here can provide guidance on evaluating the nutritive status on this uncommon species that would otherwise be difficult to obtain among wild populations.

## Graphical abstract

The current study was a comparative study of the morphology and body mass index (BMI) assessing two different geographic groups of rough‐toothed dolphins that mass stranded in South Florida, in 2004 and 2005. The focus was to determine the usefulness of the BMI during stranding triage, through rehabilitation and in determining suitable body condition level for release, to potentiate increased postrelease survival. Cover Image. Rough‐toothed dolphin (*Steno bredanensis*) in rehabilitation. Photo credit, Marine Mammal Conservancy, Key Largo, FL.

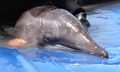

## 1 RATIONAL/BACKGROUND

Rough‐toothed dolphins (*Steno bredanensis*) are members of the family Delphinidae. They are typically found in pelagic waters throughout warmer temperate and tropical regions of the world. In the United States, there are three major recognized stocks: one in Hawaii, one in the Northern Gulf of Mexico, and one in the Western North Atlantic (NOAA Fisheries, 2012, 2014, 2017).

As a pelagic species, they are more prone to stranding than their coastal counterparts, as they are naïve to navigating the conditions of inshore waters (Geraci & Lounsbury, 2005). In addition, rough‐toothed dolphins exhibit exceptionally strong social bonding with free‐ranging social groups typically consisting of up to 50 individuals. Due to the highly communal nature of many marine mammals, individuals are inclined to remain in close proximity to one another (Jefferson, 2009; Kuczaj & Yeater, 2007; Leatherwood & Reeves, 1983; Lodi, 1992; Würsig, Jefferson, & Schmidly, 2000). As such, *S. bredanensis* have one of the highest incidences of mass strandings of any marine species, accounting for 34% of the reported mass strandings involving five or more individuals in the Southeastern United States between 1995 and 2005 (NOAA, unpublished data). The largest recorded *S. bredanensis* stranding before 2005 occurred in December of 1997 in the Florida Panhandle; it involved 62 individuals, of which only two were successfully released back into the wild (NOAA, unpublished data). Therefore, there is a clear need to be able to identify healthy individuals during triage in order to improve the outcomes associated with mass strandings of this species.

Examining “body condition” is one way of selecting the best candidates for rehabilitation. It is often defined as the amount of energy held in an individual's lipid stores (Pitt, Larivière, & Messier, 2006). These fat stores are a reliable reflection of an individual's foraging effort and success (Aguilar & Borrell, 1990). Further, morphometrics can be used as an indicator of the nutritive condition not only for the individual, but also for the entire population (Hart, Wells, & Schwacke, 2013).

Applying length‐to‐weight comparisons as an objective assessment of health is becoming common practice in cetaceans (Perrin, Dolar, Chan, & Chivers, 2005), though further research is required to improve precision and species‐specific reference ranges. There is currently no consensus on the best morphometric index with which to calculate body condition, let alone what healthy ranges look like (Kershaw, Sherrill, Davidson, Brownlow, & Hall, 2017). In a recent study, the length‐to‐girth ratio for stranded common dolphins (*Delphinus delphis*) was found to be significantly different between animals with postrelease success and failure; whereas, the length‐to‐weight ratio was not found to be a good prognostic indicator (Sharp et al., 2014). The lack of standardized morphometric ranges is a significant limitation on the clinical care and rehabilitation of rough‐toothed dolphins.

With limited resources, funding, and time veterinarians and responders often need to make decisions regarding triage, logistics, rehabilitation facilities, and animal survival prognosis based on limited clinical data. A species‐specific range for body mass index (BMI) is a useful guide during triage, when determining if an animal in rehabilitation is receiving adequate nutritional support, and/or if an animal is ready for release into the wild. Therefore, the objectives of this study were to compare BMI data from two rough‐toothed dolphin mass stranding events for differences and determine the usefulness of BMI during triage, through rehabilitation in an effort to identify a suitable body condition level, and to potentiate increased postrelease success.

## 2 METHODS

Mass stranding responses were conducted cooperatively by members of the Southeast Marine Mammal Stranding Network. Rehabilitation of stranded individuals was facilitated by the Marine Mammal Conservency, the Florida Keys Marine Mammal Rescue Team, Mote Marine Laboratory and Aquarium, and the Marine Animal Rescue Society. Postmortem examinations were performed by staff of the Florida Marine Mammal Pathobiology Laboratory, the University of North Carolina at Wilmington, the University of Tennessee College of Veterinary Medicine, the NOAA Fisheries Miami and Beaufort Laboratories, and the NOAA Office of Protected Resources.

Weights for animals were obtained using a digital hanging scale. From the scale, chains or straps supported a sling which was tared and subsequently held either a living or deceased animal. Some carcasses were suspended on a hook by the dorsal fin to get a weight reading depending on equipment availability.

Standard protocols were used to ensure that morphologic measurements were taken in a consistent manner. Length was taken by measuring the straight line distance from the tip of the snout to the notch of the flukes; while blubber thickness was measured along the maximum girth, anterior to the dorsal fin insertion, transecting at dorsal midline, midlateral, and ventral midline areas (Geraci & Lounsbury, 2005).

**Figure 1:**
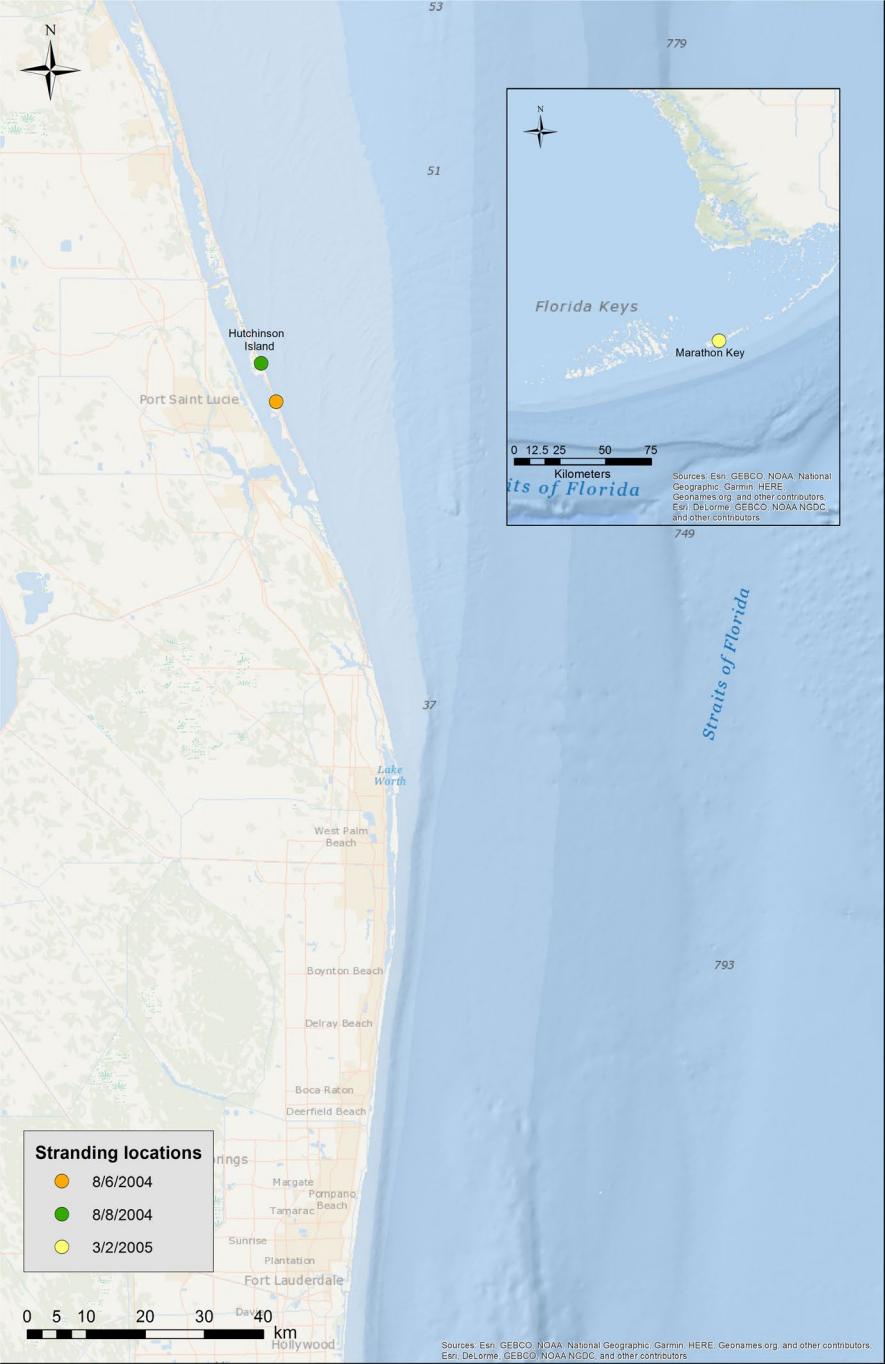
*Steno bredanensis* mass stranding locations for 2004 (orange, green) and 2005 (yellow). The majority of 2004 animals stranded August 6 and a lone animal stranded August 8

The first stranding event occurred on August 6, 2004; 37 rough‐toothed dolphins were initially found on a beach on Hutchinson Island in St. Lucie (Figure 1). During the response animals in hand were assessed, five were given dorsal fin roto‐tags before impending severe weather forced rescuers to release the animals offshore and take shelter. Nearly 3 hr later, 36 animals including the five‐tagged individuals were found restranded on a nearby beach while one was recovered alive on August 8. Of the 37 original animals, 30 were humanely euthanized on the day of the stranding. The remaining animals were moved into rehabilitation on August 9, where one died spontaneously 3 days later, three others died spontaneously over 2 months, leaving three which were successfully rehabilitated and released (Table 1).

**Table 1:** Comparison of individual demographic and morphometric variables between 2004 and 2005 *Steno bredanensis* mass stranding events

	2004	2005	*p*‐Value
Total, *n*	36^a^	32^b^	
Sex			
Male	18 (50%)	7 (21.9%)	.02
Female	18 (50%)	25 (78.1%)	
Age class			
Adult	19 (57.6%)	22 (81.5%)	.13*
Subadult	10 (30.3%)	4 (14.8%)	
Calf	4 (12.1%)	1 (3.7%)	
Unknown	3	5	
Body condition			
Underweight	3 (100%)	20 (80.0%)	.41*
Healthy weight	0	5 (20.0%)	
Unknown	33	7	
Intake weight (kg)	100.81** (*SD* = ±27.12)	137.26** (*SD* = ±25.06)	<.01
Last weight (kg)	105.17 (*SD* = ±17.52)	150.64** (*SD* = ±15.48)	<.01
Intake length (cm)	219.88** (*SD* = ±14.03)	243.48** (*SD* = ±11.13)	<.01
Last length (cm)	217.88 (*SD* = ±14.76)	241.53 (*SD* = ±9.83)	<.01
Blubber thickness			
Dorsal	1.58 (*SD* = ±0.28)	1.55 (*SD* = ±0.57)	.86
Lateral	0.82 (*SD* = ±0.25)	1.08 (*SD* = ±0.46)	.52
Ventral	0.81 (*SD* = ±0.27)	1.19 (*SD* = ±0.64)	.04
BMI			
Intake	0.45** (*SD* = ±0.1)	0.57** (*SD* = ±0.065)	<.01
Lowest	N/A	0.474 (*SD* = ±0.063)	N/A
Highest	N/A	0.646 (*SD* = ±0.044)	N/A
Last (release)	0.48 (*SD* = ±0.06)	0.62 (*SD* = ±0.05)	<.01
Pregnant females			
Intake BMI	0.58 (*SD* = N/A) (*n* = 1)	0.59 (*SD* = ±0.03) (*n* = 2)	.94
Lactating females			
Intake BMI	N/A	0.53 (*SD* = ±0.065) (*n* = 2)	N/A
Outcome			
Euthanized	29 (80.6%)	5 (15.6%)	<.01
Natural death	4 (11.1%)	15 (46.9%)	<.01
Released	3 (8.3%)	11** (34.4%)	<.01
Change in BMI	0.06 (*SD* = ±0.06)	0.07 (*SD* = ±0.53)	.73
Unadjusted intake BMI by outcome			
Euthanized	0.43 (*SD* = ±0.14)	0.548 (*SD* = ±0.075)	<.01
Natural death	0.38 (*SD* = ±0.08)	0.585 (*SD* = ±0.063)	<.01
Released	0.44 (*SD* = ±0.11)	0.551** (*SD* = ±0.063)	.04

Abbreviation: BMI, body mass index.

a 36 of 37 animals had both length and weight recorded.

b 32 of 69 animals had both length and weight recorded.

* Unknown category was excluded from statistical comparison.

** Data from calves excluded.

In the second event, on March 2, 2005, 69 rough‐toothed dolphins stranded off Marathon in the Florida Keys (Figure 1). 37 animals died at the scene and 32 were transported to multiple rehabilitation facilities. Of those, 20 animals died in rehabilitation, 11 were successfully released back into the wild, and one calf was transferred to a marine park after being deemed nonreleasable by NOAA NMFS. An additional, 11 *S. bredanensis* carcasses were identified on West Bahia Honda Key, approximately 23 km west of the Marathon Key stranding, on March 25, 2005. It was estimated that their time of death was in line with the March 2 stranding (MMHSRP (Marine Mammal Health and Stranding Response Program), 2017), indicating that they were potentially from the same pod as the animals that were found on March 2. Due to the advanced degree of decomposition, the carcasses were not brought in for necropsy and no additional data were obtained.

Data were obtained from NOAA as well as the MMHSRP online database for each stranding event. Records from the events were evaluated and individuals with both length and weight measurements were selected for the study. In the 2004 stranding, 36 of 37 had both length and weight measurements recorded while 32 of 69 from the 2005 event had complete data. Eleven adult animals from the 2005 stranding had lengths and weights recorded periodically throughout their rehabilitation process for analysis.

### 2.1 Statistical analysis

Body mass index was calculated using methods published by Kershaw et al. (2017) comparing morphometric equations for body mass index in small cetaceans suggesting that normalizing the data can be achieved using a simple calculation:

$$\text{BMI}=\frac{\text{weight}\;\left(\text{in}\;\text{kilograms}\right)}{\text{length}\;\left(\text{in}\;\text{centimeters}\right)}$$

Comparison of gender, age class, body condition, and outcome was compared between stranding events using a chi‐square test. For continuous morphometric data (length, weight, blubber thickness, and calculated BMI), variables were assessed for normality of distribution prior to comparison using a two‐sample *t* test and ANOVA when appropriate. A general linear model (GLM) was used to compare mean BMI for each stranding event and control for differences in gender and age class. Adjusted marginal means were calculated and compared using least square differences post hoc test. Similarly, a GLM was used to compare BMI by outcome (natural death, euthanasia, release) while adjusting for age class and gender. All analyses were done using SPSS version 25 (IBM) and a *p*‐value of <.05 was considered a statistically significant result.

## 3 RESULTS

To test whether the animals in this study exhibit sexual dimorphism, a two‐sample *t* test was performed with data from all individuals by age class. These animals did not appear to be sexually dimorphic (subadults *p* = .81; adults *p* = .18), and as such, male and female individuals were grouped together in subsequent statistical calculations.

A total of 68 individuals had complete demographic and morphometric data and were included in the statistical analysis (36 in 2004 and 32 in 2005; Table 1). The 2005 stranding was comprised mostly of females (78.1%) while the 2004 stranding was split evenly between males and females (50% each). There were two pregnant and two lactating females in the 2004 stranding group and one pregnant female in 2005. The 2005 stranding had a larger proportion of sexually mature adults (81.5%) than the 2004 stranding (57.6%) (sexual maturity determined through necropsy); however, the 2004 stranding included four calves while 2005 only had one (Table 1). When comparing events, animals in the 2004 stranding were much smaller, both in intake length (*p* < .01) and weight (*p* < .01) compared to those in the 2005 event. Of the three blubber thickness measurements taken (dorsal, lateral, and ventral), only the ventral thicknesses were significantly different between the 2 years (*p* = .03), with the 2004 animals being much thinner overall. This was echoed in the intake BMI measurements, as the unadjusted average BMI at the time of stranding in 2005 was 0.13 higher than that in 2004 (Table 1). After adjusting for differences in age class and gender, the mean BMI for 2005 remained significantly (*p* < .01) higher (0.41; Std Error [*SE*] = 0.02) than animals stranding in 2004 (0.34; *SE* = 0.02).

According to necropsy reports, Cetacean Data Records, and notes from the stranding events themselves, three individuals were described as being “underweight” from the 2004 stranding; 20 from 2005 were “underweight” while five were described as “healthy weight.” This is, however, a largely subjective measurement and subject to observer bias. Notes from the 2004 stranding event were very sparse, with no other information on apparent body condition besides the three “underweight” individuals, leaving 33 animals unknown.

**Figure 2:**
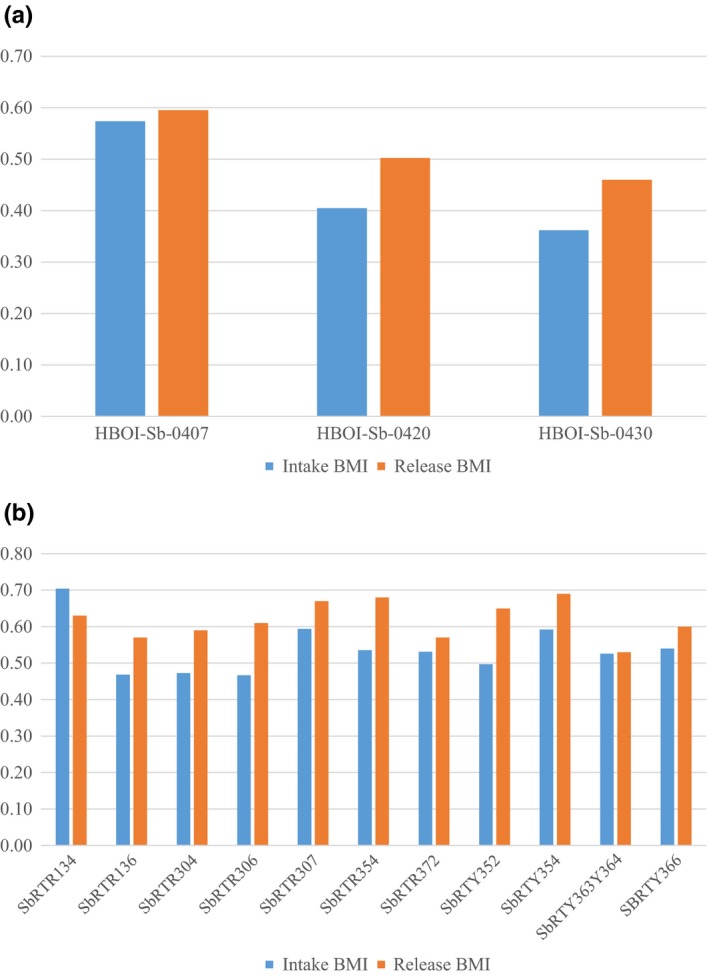
Comparison of individual *Steno* intake and release body mass index (BMI) for the (a) 2004 and (b) 2005 stranding events

The data for the 2004 stranding show that five animals (with both a recorded intake and release/necropsy weight) gained weight during rehabilitation (Figure 2a). Individual weight gain was observed among all animals with a maximum BMI increase of 0.16 (27.5 kg) for individual HBOI‐Sb‐0410. However, the difference between intake and release BMI was not significant (*p* = .395) for the group overall. The 2005 stranding population showed a statistically significant difference between intake and release BMI (*p* < .01) with all but one animal gaining weight during rehabilitation (Figure 2b). The animals that underwent rehabilitation from each stranding group gained, on average, a comparable amount of weight in relation to their length (0.06 average increase in 2004; 0.07 average increase in 2005).

For the 2004 population, there were no physical parameters associated with survival. In the 2005 population, both intake length (*p* = .038) and intake weight (*p* = .03) were significantly associated with survival. In both cases, a smaller value was correlated with a higher rate of survival. The age and gender‐adjusted mean (*SE*) intake BMI's by outcome were 0.36 (*SE* = 0.02) for euthanized individuals, 0.48 (*SE* = 0.02) for natural death and 0.51 (*SE* = 0.03) for dolphins that were successfully released. The mean BMI for individuals that were released was significantly higher than dolphins that were euthanized (*p* = .03) but not significantly higher than natural deaths (*p* = .56). Similarly, individuals who died naturally had a significantly higher (*p* < .01) mean BMI on intake compared to dolphins that were euthanized.

**Figure 3:**
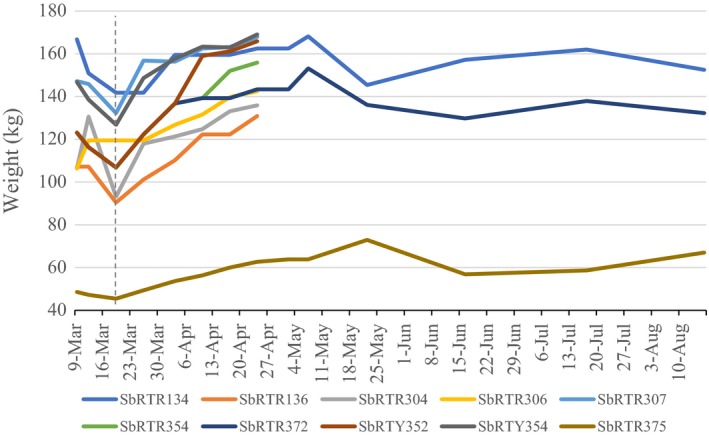
Individual *Steno* weights through rehabilitation from 2005 stranding event (dotted line denotes March 19, 2005)

Information regarding weight throughout the rehabilitation process was available for most of the animals from the 2005 stranding. Individual weights were taken periodically and recorded in each animal's rehabilitation file (Figure 3). Almost all the animals show an early sharp drop in weight leading up to March 19. This was likely due to the initial implementation of a bottlenose dolphin (*Tursiops truncatus*) diet and feeding schedule (see Section 4). At the time of the 2005 stranding event, there were no established feeding protocols for rough‐toothed dolphins, which resulted in using dietary regiments commonly used for bottlenose dolphins. As a result, the diet was modified, and all the animals began to gain weight following March 19.

**Figure 4:**
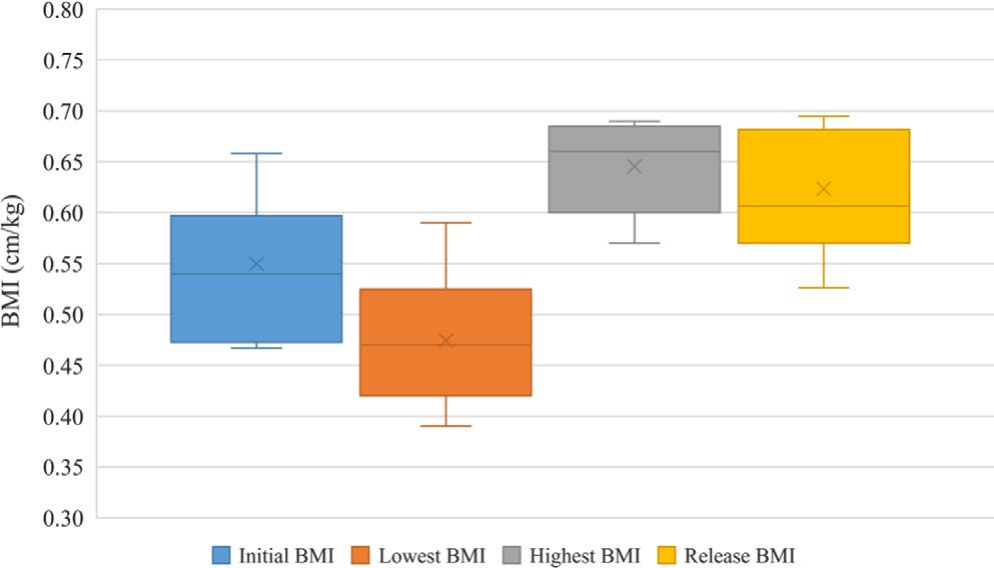
Group changes in body mass index (BMI) from intake to release for 2005 *Steno* mass stranding event. *x* = mean; center line = median

Ultimately, the animals in rehabilitation from 2005 had three separate release dates, which were dependent on when individuals were found to be in good body condition and clinically stable for release (Figure 4). The first group was released on April 20, 2005 with release BMI values of 0.53 and 0.60. The second group was released on May 3, 2005, with release BMI of 0.57, 0.59, 0.61, 0.65, 0.67, 0.68, and 0.69. The last two individuals had remained in rehabilitation for a total of 195 days before being declared clinically stable and ready for release with BMI values of 0.57 and 0.63 on September 12, 2005 (Figure 2b). Animals in rehabilitation for 2004 were found to be in good body conditions, clinically stable, and were released together with BMI values of 0.46, 0.50, and 0.60 on March 3, 2005, after a total of 210 days (Figure 2a).

## 4 DISCUSSION

In this study, we compared BMI across stranding outcomes for rough‐toothed dolphins. There was evidence of differences by geographic location, suggesting that the two populations (Atlantic and Gulf of Mexico) may be more distantly related than previously assumed. Further, there was no sexual dimorphism evident in either population (*p* = .99), indicating that differences in morphology were not caused by the sex of the stranded individuals (it should be noted; however, that several studies have found evidence of sexual dimorphism in this species, Miyazaki & Perrin, 1994; West, 2002).

Mortality in a stranding situation is a function of physical trauma, preexisting health status, and overall dispersal of individuals in the stranding event (Sampson et al., 2012). Commonly noted clinical signs in marine mammal strandings include abnormally rapid breathing, abnormally high resting heart rate, vomiting, body arching or thrashing, vocalization, and loss of reflexes (Sampson et al., 2012), likely a result at least in part from the physical trauma of being beached and the high thermal stress in South Florida. In fact, many postmortem pathological studies suggest the presence of disease/disorder(s) existing before the stranding event itself (Cowan, 2009).

Visual assessment of nutritive status is utilized to evaluate both wild and captive animal populations (Joblon et al., 2014; Pettis et al., 2004). BMI uses morphologic measurements to put values to visual assessments, thereby giving a quantitative rather than qualitative measure. Body mass index is important for measuring health status, but it can be deceiving; therefore, a BMI calculation should always be accompanied by a physical examination and/or visual assessment, and vice versa. The two work hand in hand to give a complete evaluation of an animal's health using both subjective and objective components. Further, knowing where the animal stranded (being potentially indicative of stock origin [i.e., Atlantic vs. Gulf of Mexico]) can allow for better interpretation of assessment measures. We have shown that there were differences in size and morphology even after adjusting for differences in age class and gender in that the mean BMI for 2005 remained significantly higher than for the animals stranding in 2004.

For veterinarians, weight is an important metric for calculating dosages of certain medications, including euthanasia drugs. The effects of many medications are dose‐dependent, making accurate weight estimation essential to treatment or euthanasia to prevent under‐ or overdosing the animal (Tasker, 2008). Barco et al. established weight approximations based on length‐weight regressions in 2012 for several marine mammal species to facilitate calculating euthanasia drug dosages in the field. The weight approximations were calculated for various species including bottlenose dolphins (*T. truncatus*) and striped dolphins (*Stenella coeruleoalba*); however, no estimations were made for rough‐toothed dolphins (Barco et al., 2012).

Calculating the BMI for wild, non‐stranded individuals requires the capture and weighing of free‐swimming animals. Several methods have been tested for estimating nutritive status in the wild; comparing total length to maximum width has shown promising results in estimating both reproductive and nutritive status in large free‐swimming cetaceans (Perryman & Lynn, 2002; Pettis et al., 2004). Physical indicators can also be utilized to assess overall health and nutrition. In domestic mammals, including cows, horses, and dogs, there are specific morphologic points (e.g., spinal processes, ribs, hips) that can be evaluated to create a general, qualitative measure (Eversole, Browne, Hall, & Dietz, 2009). Joblon et al. (2014) developed a body condition scoring system for assessing the nutritional status of delphinids using anatomical landmarks, which were indicative of body condition and emaciation. Visual assessment can be very useful in determining body condition in free‐swimming populations and during stranding response triage.

Understanding patterns of fat mobilization are vital to making proper visual assessments of an individual animal's health status. Studies on blubber distribution have shown that, during periods of starvation, blubber is mobilized almost exclusively from the thorax while tailstock blubber is unaffected, suggesting that blubber caudal to the anus functions exclusively in locomotion and is metabolically inert (Koopman, Pabst, McLellan, Dillaman, & Read, 2002). In harbor porpoises (*Phocoena phocoena*), thoracic blubber thickness in emaciated animals was found to be half of that in healthy animals, while tailstock blubber showed no difference in thickness. As such, there is no “one size fits all” definition of body condition; species‐specific descriptions are necessary to most accurately predict an individual's overall health status.

One morphological parameter, dorsal blubber thickness, was not statistically different between the two mass stranding events. In delphinids, the dorsal blubber thickness is largely unimpacted in cases of acute emaciation (NOAA, personal communication), suggesting that the two populations were not in a state of chronic emaciation in which fat stores along the epaxial musculature would be mobilized. The significant difference between the lateral and ventral blubber thicknesses suggests these measurement sites could be reference points for determining body condition. Thinning of the lateral blubber layer (i.e., thoracic body wall), results an increased visibility of the ribs with declining body condition defining emaciation (Joblon et al., 2014).

Using morphometrics as positive prognostic indicator for determining potential rehabilitation success could have a significant impact on the triage and rehabilitation process, when resources are sparse, and efforts are best utilized for animals with the greatest chances of being released. A 2014 study suggested that failed animals in rehabilitation had significantly higher length‐to‐girth ratios that those that were released (Sharp et al., 2014), indicating that thinner animals were less likely to survive. In this study, only two parameters in the 2005 population proved to be connected to survival: a smaller intake length (*p* = .038) and a smaller intake weight (*p* = .03). This indicates that younger, smaller animals had the greatest chances of surviving the initial stranding event as well as succeeding in rehabilitation. In a mass stranding event, the younger naïve animals tend to be following the older individuals, suggesting that they may be in better health than larger animals that are stranding due to health problems. Intake length and intake weight were not significantly related to survival outcome for the 2004 group, and intake BMI was not significantly related to survival outcome for the 2004 or 2005 populations, respectively.

Data from satellite tags help to better illustrate the long‐term success of these animals after being released. VHF and Splash transmitters on the three animals from 2004 successfully tracked individuals HBOI‐Sb‐0420 and HBOI‐Sb‐0407 with transmission data for 20 and 23 days, respectively, while individual HBOI‐Sb‐0430 only had 3 days of data. Five animals released from the 2005 stranding event were tagged with satellite‐linked transmitters: The transmission lengths were 12, 14, 30, 38, and 49 days (Wells, Early, Gannon, Lingenfelser, & Sweeney, 2008). One tag (on individual SbRTR366) exceeded the expected number of transmissions by 37%. On May 7, 2005, a helicopter photographed seven of the released animals traveling together. Tags on the two individuals with the least number of transmissions (SbRTR134 and SbRTR372) stopped transmitting data shortly after Hurricane Rita, September 20, 2005. While the official measure for a success rehabilitation/release was set at 4 weeks, a duration that only three individuals were proven to have successfully achieved, all 10 of the tagged released animals were determined to be successfully rehabilitated (Wells et al., 2008).

Body mass index comparisons found in this study may be utilized in rehabilitation settings to help determine when individuals are at a healthy weight and potentially ready for release. According to data from the 2005 stranding event, consequential rehabilitation and postrelease satellite monitoring, individuals found to be clinically stable, in good body condition and deemed ready for release with a BMI >0.53 were successfully rehabilitated. For the 2004 animals, according to postrelease satellite monitoring, only animals with a BMI above 0.5 had adequate transmission data to be considered successfully released (Manire & Wells, 2005).

In the case of the 2005 stranding animals that entered rehabilitation, no published diet information was available for *S. bredanensis*; consequently, they were fed using a common bottlenose dolphin (*T. truncatus*) diet used in captivity (consisting largely of capelin [*Mallotus villosus*], herring [*Clupea* spp.], and squid [*Sepioteuthis sepioidea*]) and feeding schedule (Marine Mammal Conservancy, unpublished notes). This resulted in immediate dramatic weight loss in all but one individual (who remained at a stable weight) until the diet was altered. Further, this original diet caused several of the animals to develop acute pancreatitis (NOAA, unpublished data). As such, their diet was reconfigured to include less fat and more protein, with an overall increase in the total weight of fish given per day, resulting in improved BMI (Figure 3).

In the future, consistent collection of weight and length measurements during rehabilitation efforts by facilities could improve BMI estimates for rough‐toothed dolphins along with the consistent collection of maximum girth measurements and blubber thicknesses. Evaluation of an individual's overall health status cannot rely solely on a BMI calculation. The initial intake BMI for the two stranding events in this study was significantly different, suggesting the two populations were negatively impacted by different mechanisms and/or for different time periods prior to the stranding event. Furthermore, some diseases and early anorexia do not manifest with a dramatic decrease in body mass until very late stages and in those instance changes in BMI may help in monitoring animals receiving treatment. As such, BMI does not provide a complete picture of an animal's health. However, it can offer a reliable and rapid indication of severe chronic illness, emaciation and anorexia, and reproductive status when more invasive diagnostics are not an option, most notably in mass stranding situations, where rapid triage is essential for determining the best candidates for rehabilitation.

A well‐defined scale for evaluating overall body condition could be instrumental in determining whether certain practices, such as specific diet and feeding schedule, were directly benefitting or harming the animals. While body condition and body mass index measurements obtained during rehabilitation may not represent realistic expectation for wild individuals, understanding patterns in controlled environments offers some insight. In this study, we determined that BMI can be used as a prognostic indicator during rehabilitation efforts to evaluate adult rough‐toothed dolphins for release. Ultimately, this data can be utilized in the care of stranded rough‐toothed dolphins from stranding events to improve rehabilitation outcomes.

## CONFLICT OF INTEREST

None declared.

## AUTHOR CONTRIBUTIONS

All contributing authors have approved this work for publication. Individual contributions are listed as follows: Study concept and design—BLK, RYE, AMS; Data compilation, analysis and interpretation—BLK, RYE, AMS; Statistical analysis and interpretation—BLK, AMS; Manuscript preparation—BLK, RYE, AMS.

## Data Availability

The marine mammal stranding demographic and morphological data upon which this manuscript was formulated are publically available from the Dryad Digital Repository: https://doi.org/10.5061/dryad.pp419k6. Additional stranding related marine mammal data are accessible by request to the U.S. DOC, NMFS, Protected Resources Division, Marine Mammal Health and Stranding Response Program, Southeast Region reference NMFS InPort Catalog ID# 55065 https://inport.nmfs.noaa.gov/inport/item/55065.
